# Agreement and Consistency of Visual and Digital Tooth Shade Selection: An In Vivo Comparison of Expert Practitioners and Senior Dental Students

**DOI:** 10.3390/healthcare14213700

**Published:** 2026-11-01

**Authors:** Mai Soliman, Shahad Alqahtani, Lamees Alsalmah, Joud Alshahrani, Futoon Albogmi, Nouf Alajmi, Elzahraa Eldwakhly, Alhanoof Aldegheishem, Rasha Alharthi, Sarah Alghamdi

**Affiliations:** 1 Department of Clinical Dental Sciences, College of Dentistry, Princess Nourah bint Abdulrahman University, P.O. Box 84428, Riyadh 11671, Saudi Arabia; 2 Dental Intern Student, College of Dentistry, Princess Nourah bint Abdulrahman University, P.O. Box 84428, Riyadh 11671, Saudi Arabia

**Keywords:** spectrophotometers, shade matching, Rayplicker, tooth color, color measurement

## Abstract

**Background:** Accurate shade selection is essential in restorative and prosthetic dentistry, but visual matching is subjective and influenced by lighting, clinical experience, and color perception. Digital spectrophotometers offer objective, reproducible measurements. **Objective:** The aim of this study was to compare visual shade selection using the VITA 3D-Master guide by expert practitioners and senior dental students against a digital spectrophotometer (Rayplicker Handy), and to assess inter-examiner consistency within each group. **Methods:** Five expert practitioners and five senior dental students selected shades (cervical, middle, and incisal thirds) on the maxillary right central incisor in 40 participants, followed by digital measurement. Shade values were converted to CIE Lab* coordinates to compare groups, evaluate inter-examiner consistency, and calculate intraclass correlation coefficients (ICC). **Results:** Expert–student agreement was close, with small mean ΔE (2.1–2.5 across regions), above the perceptibility threshold (1.2) but within the acceptability threshold and no significant difference (Friedman test, Dunn’s post hoc, *p* > 0.05 in all regions). Both groups differed markedly from the Rayplicker (mean ΔE 11.9–16.2), exceeding the 50:50% acceptability threshold (ΔE*ab = 2.7) and significantly greater than the expert–student difference (*p* < 0.001); expert–Rayplicker and student–Rayplicker differences did not differ from each other (*p* > 0.05). Inter-examiner within-group dispersion was significantly lower (better agreement) among experts than students in the incisal (*p* < 0.001) and cervical (*p* = 0.049) thirds, with no difference in the middle third (*p* = 0.116); intraclass correlation coefficients were directionally higher for experts in all nine region-by-component comparisons, though confidence intervals overlapped between groups and absolute reliability was poor-to-moderate for both. **Conclusions:** Visual and digital shade selections differed substantially in this study, exceeding the clinically acceptable 50:50% threshold, while expert practitioners and senior students chose comparably close shades to one another using the visual method. However, experts were more internally consistent than students in most regions, suggesting that clinical experience was associated with greater reproducibility, though not necessarily accuracy, of visual shade selection.

## 1. Introduction

Shade matching is a complex and challenging procedure because of the many factors that influence its accuracy and reproducibility [1]. Variables such as the type and intensity of the light illuminating the tooth surface, the optical properties of dental tissues, and individual visual perception can significantly affect shade selection [2]. In addition, inconsistencies in communication between the dentist and the dental technician may further contribute to discrepancies in achieving the desired shade.

The accurate identification and reproduction of natural tooth color represent a persistent and significant challenge in restorative and aesthetic dentistry, critical for successful clinical restoration, particularly for aesthetics [3]. This inherent difficulty stems from the complex nature of color perception itself, which is influenced by the intrinsic biological properties of teeth as well as external factors such as lighting conditions [3].

Human color perception is inherently subjective, resulting from complex physiological and psychological responses that vary significantly between individuals and can also differ for the same individual over time [4]. The brain interprets the color, which can lead to differences even when two individuals view the same shade, as eyes might pick up the color, but it is the brain that interprets it [5]. Further complicating this perception are factors such as eye fatigue, mood, and even potential color blindness in the observer; older dentists may experience diminishing eyesight or have color blindness, directly affecting their ability to distinguish hues [6]. Beyond the observer, natural teeth are not monochromatic; they exhibit a complex interplay of shades, transparencies, and nuances, and capturing the full spectrum of these variations is essential for a seamless match [7]. This polychromatic nature arises from the layered structure of enamel and dentin, where the transparency of enamel interacts with the underlying yellowish dentin, making visual selection particularly challenging due to this double-layering effect [8].

The same tooth can appear differently under various light sources, such as incandescent versus natural daylight, due to their distinct spectral distributions; if the light source changes, the light reflected from an object also changes, leading to a different perceived color [9]. This phenomenon, known as metamerism, where two colors appear to match under one lighting condition but not under another, occurs when the relationship between light, object, and observer changes and can significantly compromise shade-matching efforts if not accounted for [9]. Environmental factors like surrounding wall colors or the color of adjacent teeth and gums can also influence perceived shade due to reflection and contrast effects; brightly colored walls or clothing can reflect onto the tooth, creating false color perceptions [3].

This fundamental challenge of identifying the “true” tooth color arises from a complex interplay where the inherent subjectivity of human color perception is compounded by the intrinsic biological variability and polychromatic nature of natural dentition, which is then further distorted by extrinsic environmental factors. This multi-layered causality suggests that addressing only one aspect, such as standardizing light, will not fully resolve the problem without considering the others, leading to significant clinical and economic consequences such as patient dissatisfaction and costly re-dos, often necessitating re-doing the procedure, which incurs additional time and expense for both the patient and the dentist [3].

A key distinction between conventional visual and digital shade selection techniques lies in their reproducibility and susceptibility to examiner-related factors. The human eye is influenced by the lighting conditions, the examiner’s level of experience, fatigue, and the unique characteristics of the tooth, all of which can lead to variability in shade selection [2,10]. In contrast, standardized conditions provided by digital shade-matching systems can improve consistency and enhance communication between clinician and technician, ultimately supporting more accurate results and higher patient satisfaction [10,11].

Digital shade selection incorporates advanced optical devices and software—often integrating artificial intelligence and machine learning—to analyze tooth color and determine hue, chroma, and value with greater precision [3]. Unlike conventional methods that rely primarily on subjective visual comparison and are influenced by environmental and operator-related factors, digital systems such as spectrophotometers, colorimeters, and intraoral scanners provide objective and standardized measurements [4,5]. By capturing reflected light from the tooth surface and processing it through calibrated sensors and algorithms, these systems reduce inconsistencies and improve the reproducibility of shade selection [5,6,12].

Precision in shade selection plays a critical role in achieving satisfactory esthetic outcomes and patient acceptance of restorations [7]. Conventional shade guides, such as the Vita 3D-Master (VITA Zahnfabrik, Bad Säckingen, Germany), are widely used; however, their performance is inherently subjective and affected by lighting conditions and the clinician’s experience [7]. In contrast, digital shade-matching devices, such as the Rayplicker Handy (Borea, France), provide a standardized acquisition protocol combined with dedicated software, offering results that are less dependent on the operator and more easily reproducible [8]. Recent studies indicate that digital shade-matching systems can enhance both the precision and reliability of shade determination compared with conventional visual methods [8,13]. Unlike spot-measuring spectrophotometers, the Rayplicker Handy captures the whole labial surface in a single image and reports color separately for the cervical, middle and incisal thirds; it has also shown close agreement with a reference spectrophotometer in vivo [14].

VITA produces several visual shade guides. The VITA classical (VITAPAN classical) guide, introduced in the 1950s, has 16 tabs grouped by hue into four families: A (reddish-brown), B (reddish-yellow), C (gray) and D (reddish-gray), with numbers indicating increasing chroma and darkness. Its tabs were derived empirically and are unevenly distributed in color space [15,16]. The VITA Tooth guide 3D-Master was developed to represent the natural tooth color space more systematically. Its 29 tabs (26 standard shades plus three bleached shades) are arranged in six lightness groups, each subdivided by chroma and hue (L = yellowish, M = medium, R = reddish), so that shade is selected in the order lightness, chroma, hue [16]. The VITA Linear guide 3D-Master holds the same 29 shades in a linear format: lightness is selected first from a dedicated value guide, then chroma and hue from the corresponding tab holder, which simplifies selection. A comparative study found a lower coverage error for the 3D-Master system than for VITA classical (mean ΔE*ab 3.93 vs. 5.39) [16]. Visual coverage errors for both guides nevertheless exceed the 50:50% acceptability threshold [15,17], and only about 40% of natural tooth colors correspond closely to a 3D-Master tab [18]. The VITA Tooth guide 3D-Master was therefore chosen for this study as the most systematically designed of these guides, although even this guide cannot reproduce every natural tooth color.

Therefore, this study aims to evaluate and compare the agreement and consistency of tooth shade selections made by expert dental practitioners and senior dental students using both a conventional visual method (Vita 3D-Master shade guide) and a digital shade-matching device (Rayplicker Handy, Borea, Saint-Jean-d’Illac, France). Shade selection will be assessed at the cervical, middle, and incisal thirds of the maxillary central incisor to determine potential differences in performance between techniques and between levels of clinical experience.

## 2. Materials and Methods

This in vivo study received ethical approval from the Institutional Review Board of the Deanship of Scientific Research, Princess Nourah bint Abdulrahman University (IRB no. 25-0169). All participants provided informed consent, and procedures were conducted in accordance with relevant guidelines and regulations.

**Figure 1:**
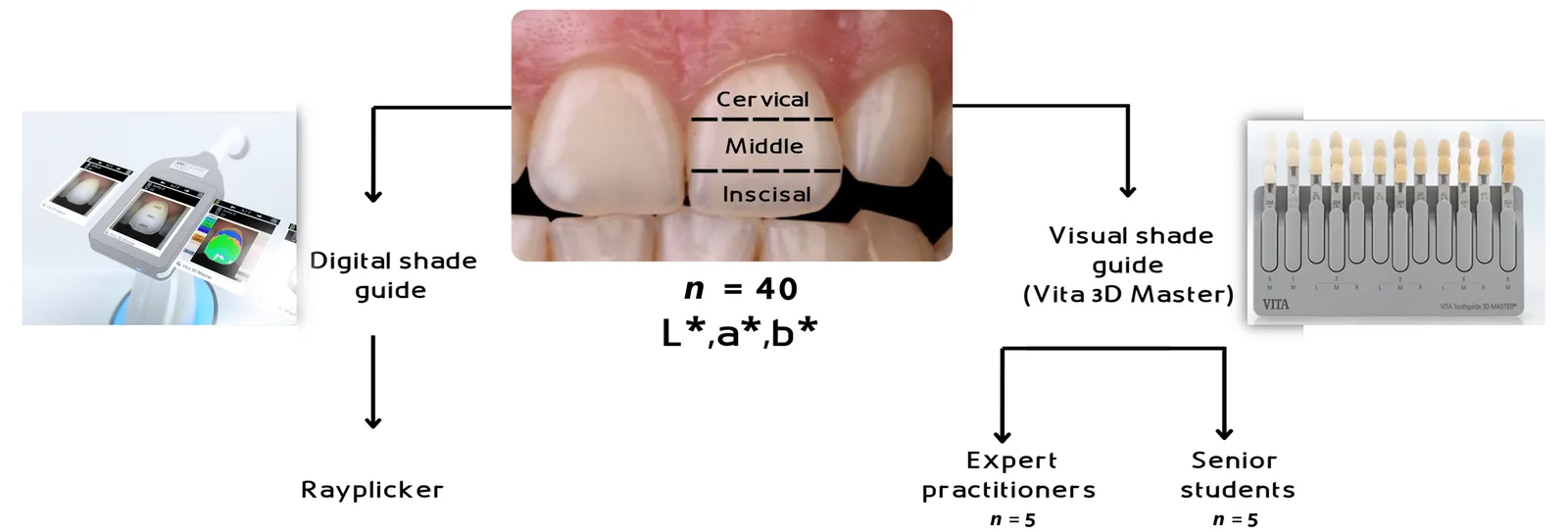
Study design. The "*" in "L*,a*,b*" is standard CIELAB notation.

Study design: This was an in vivo, cross-sectional, single-center method-comparison study with a fully crossed design, reported according to the Guidelines for Reporting Reliability and Agreement Studies (GRRAS) [19]. Each of the 40 participants was assessed by ten examiners (five expert practitioners and five senior dental students) with the VITA Tooth guide 3D-Master and then measured once with the Rayplicker Handy (Borea, France). The maxillary right central incisor was assessed in three regions (cervical, middle and incisal thirds), giving 1200 visual and 120 digital regional observations. The study made two comparisons: (i) agreement between visual and digital shade determination, and (ii) differences between examiner groups in visual shade selection and within-group dispersion. Each participant was assigned a unique code to ensure confidentiality (Figure 1).

Sample size: No a priori sample-size calculation was performed. Participants (*n* = 40) and examiners (five per experience group) were convenience samples of those eligible and available during the study period, so the comparison between experience groups is exploratory. The primary outcome was the color difference (ΔE*ab) between visual and digital shade selection in each tooth region. A sensitivity power analysis (two-sided, α = 0.05, 1 − β = 0.80) showed that the participant-level paired comparison (*n* = 40) could detect a minimum standardized effect of dz = 0.45 (dz = 0.53 at a Bonferroni-adjusted α of 0.0167), whereas the examiner-level comparison (*n* = 5 per group) could detect only very large effects (d ≈ 2.0). Retrospective (observed) power was not calculated, because it is a direct function of the obtained *p*-value and adds no information [20].

Examiner groups: Expert practitioners were defined a priori, in line with previous shade-matching research, by professional qualification (faculty consultants in restorative dentistry or prosthodontics) [21,22], at least 10 years of post-licensure clinical experience [23], and routine involvement in clinical shade selection (≥20 cases per month) [24]. Senior dental students were final-year dental students who performed shade selection only under clinical supervision; they had received formal training in color science and shade selection in the undergraduate restorative and fixed prosthodontics courses, including preclinical exercises with the VITA Tooth guide 3D-Master and supervised clinical practice. The investigators selected the examiners from eligible faculty and final-year students at the College of Dentistry, and no additional study-specific calibration was performed. All examiners were female, and none wore corrective lenses. Before data collection, all visual examiners were screened for congenital color vision deficiency with the 38-plate Ishihara pseudoisochromatic test, following the procedure of Kalyani et al. [25]. Plates were viewed under natural daylight, with 5 s allowed per plate, and color vision was classified as normal when 17 or more of plates 1–21 were read correctly, according to the test manual. All examiners had normal color vision. Examiner characteristics are summarized in Table 1.

Each examiner recorded shades for all participants using the VITA 3D-Master shade guide. Digital measurements were then obtained for the same tooth regions using the Rayplicker Handy according to the manufacturer’s instructions. All measurements were tabulated for statistical analysis.

Participants and tooth selection criteria: Participants were included if they met predefined eligibility criteria, including dental students aged 20–25 years with good oral hygiene, no active dental disease, and willingness to provide informed consent. Exclusion criteria were direct or indirect restorations (including crowns and veneers), discolorations, or developmental anomalies affecting the maxillary right central incisor; any history of professional or at-home tooth whitening; history of orthodontic treatment involving the maxillary anterior region; or systemic conditions known to affect tooth color or oral health. Tooth selection criteria require an intact maxillary right central incisor with no restorations or visible discoloration, no history of trauma or endodontic treatment, and the presence of adjacent natural teeth for proper shade comparison. Teeth with restorations, fractures, evident wear, intrinsic or extrinsic staining, or periodontal disease altering clinical appearance were excluded. Participants were female dental students from academic years other than that of the student examiners; no individual served as both a participant and an examiner.

Shade determination procedure: All sessions took place in the same clinic room and dental unit between 9:00 and 12:00, under the same overhead LED lighting (manufacturer-rated correlated color temperature 5500 K; color rendering index ≥ 90) combined with ambient window daylight. Lighting was standardized by keeping the room, light sources and session hours constant; color temperature and illuminance were not measured or verified instrumentally. Participants sat upright with the head supported and the occlusal plane parallel to the floor, with the maxillary right central incisor at the examiner’s eye level. The tooth was cleaned and kept moist with water throughout the assessments, and was not dried, to avoid dehydration-related color change. No rubber dam or lip retractor was used. A neutral-colored bib covered the participant’s clothing, examiners avoided brightly colored attire, and no additional background shield was used. Each examiner assessed the tooth individually, in randomized order, from 25–30 cm. Using the VITA Tooth guide 3D-Master, the examiner selected the shade for the cervical, middle and incisal thirds in the manufacturer’s recommended sequence: lightness group first, then chroma, then hue. The shade guide was cleaned regularly, and the selected tab was held next to the tooth for comparison. Assessment time was limited to reduce eye fatigue, and selections were recorded privately on an individual data sheet.

Blinding and examination order: Visual assessments were completed before the digital measurements, so examiners were blinded to the Rayplicker results. Each examiner assessed each participant individually, with the other examiners outside the examination room, and recorded the selected shade privately on a separate data sheet, so that no examiner could observe the selections of others. The order of participants and the order of examiners for each participant were randomized.

Digital measurements: Digital shade determination was performed with the Rayplicker Handy (Borea, Saint-Jean-d’Illac, France). According to the manufacturer, this multispectral imaging spectrocolorimeter combines a spectrophotometer (400–700 nm) with a two-dimensional optical sensor to record color across the whole labial surface [26]. A single trained operator, blinded to the examiners’ visual selections, performed all measurements after the visual assessments for that participant were complete. The device was calibrated according to the manufacturer’s protocol at the start of each session, using its dedicated autoclavable calibration tip. With the tooth surface kept moist, the operator placed the measuring tip against the labial surface of the maxillary right central incisor; this contact fixes the working distance and angle and excludes ambient light. One image was acquired per participant. Because this image covers the whole labial surface, the software defined the cervical, middle and incisal thirds on it without repositioning the device, and the L*a*b* values for each third were extracted with the device software.

Conversion of visual shade selections to CIELAB: Each VITA 3D-Master shade designation selected by the examiners was converted to CIELAB coordinates using the fixed L*a*b* values published by Bayindir et al. [16]. These were measured with a spectroradiometer (380–780 nm at 2 nm intervals; 45°/0° illumination/viewing geometry; central 1 mm area of each tab, without backing) and reported for CIE illuminant D65 and the CIE 1931 2° standard observer. The same triplet was assigned to every selection of a given tab across all examiners and participants. The CIELAB color difference was calculated as ΔE*ab = [(ΔL*)^2^ + (Δa*)^2^ + (Δb*)^2^]^½.

Statistical analysis: Data were analyzed using IBM SPSS Statistics for Windows, Version 20.0 (IBM Corp., Armonk, NY, USA). Normality was assessed using the Shapiro–Wilk test. Continuous variables were summarized as mean ± standard deviation for normally distributed data and as median (interquartile range) otherwise. Color differences were calculated using the CIELAB ΔE*ab formula, consistent with other recent comparable visual-versus-digital shade-matching studies [18,21,27,28]. Results were interpreted against the 50:50% perceptibility threshold (PT; ΔE*ab = 1.2, ΔE00 = 0.8), the difference detected by 50% of observers, and the 50:50% acceptability threshold (AT; ΔE*ab = 2.7, ΔE00 = 1.8), the difference judged clinically acceptable by 50% of observers [17,29]. These thresholds are population averages obtained under controlled viewing conditions and were used as interpretive benchmarks, not as absolute clinical limits. Comparisons across methods were performed using the Friedman test for related samples, followed by Dunn’s post hoc test for pairwise comparisons. Statistical significance was set at *p* ≤ 0.05. As a supplementary sensitivity analysis, inter-examiner dispersion for each tooth region was modeled using a linear mixed-effects model with participant and examiner specified as crossed random effects and group (expert/student) as a fixed effect, to directly test whether the group effect persisted after accounting for examiner-level clustering [30,31]. Given the three regional comparisons performed for inter-examiner within-group dispersion, a Holm–Bonferroni correction was applied across regions for both analyses to control the family-wise error rate [32]. A two-way random-effects, absolute-agreement intraclass correlation coefficient (ICC) was calculated for each tooth region and CIE L*a*b* component within each examiner group, using the single-measures (ICC(A,1)) and average-measures (ICC(A,k), k = 5 examiners) forms with 95% confidence intervals [33].

## 3. Results

Forty participants were evaluated by 10 examiners (five expert practitioners and five senior dental students) using both visual (Vita 3D-Master shade guide) and digital (Rayplicker Handy) shade-matching techniques. Shade measurements were recorded separately for the cervical, middle, and incisal thirds, and color data were analyzed in the CIELAB color space using L* (lightness), a* (red–green axis), and b* (yellow–blue axis) values (Table 2).

**Figure 2:**
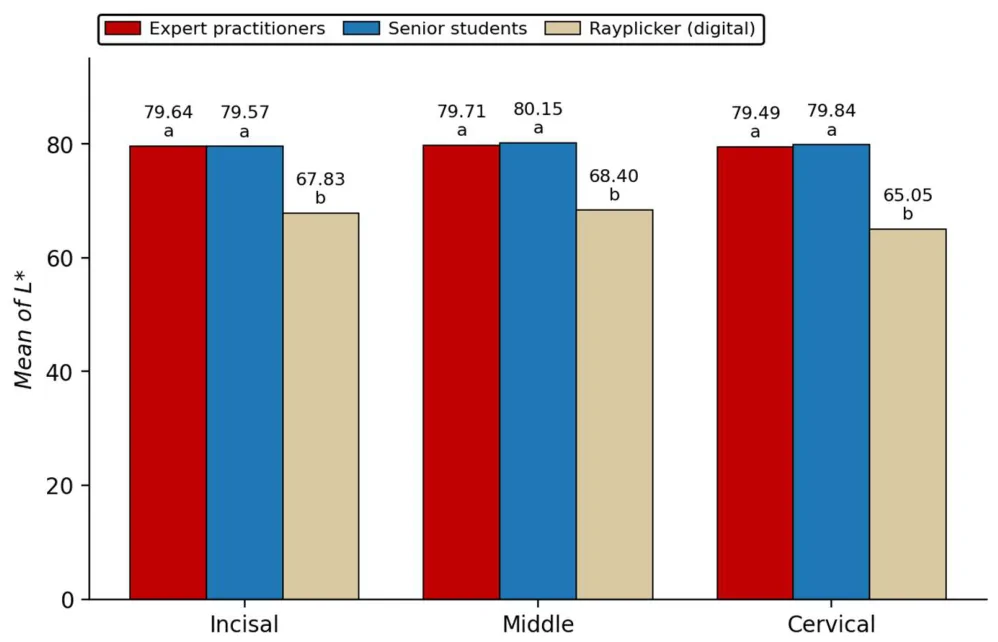
L* Comparison between Expert practitioners, senior students and L* in each location (*n* = 40). Different letters above bars indicate statistically significant differences between groups (Dunn’s post hoc test), *p* ≤ 0.05.

For L* (lightness) values, no significant difference was observed between expert practitioners and senior students across all tooth thirds (*p* > 0.05), indicating comparable lightness perception between the two examiner groups (Figure 2). In contrast, the Rayplicker Handy recorded significantly lower L* values, corresponding to darker shades, compared to both human groups in the incisal, middle, and cervical thirds (*p* < 0.001 for all comparisons). Overall, the Friedman test revealed statistically significant differences among the three methods in all regions (*p* < 0.001), and post hoc analysis confirmed that experts and students produced closely matched scores (*p* > 0.05), while Rayplicker measurements were consistently lower (*p* < 0.001).

**Figure 3:**
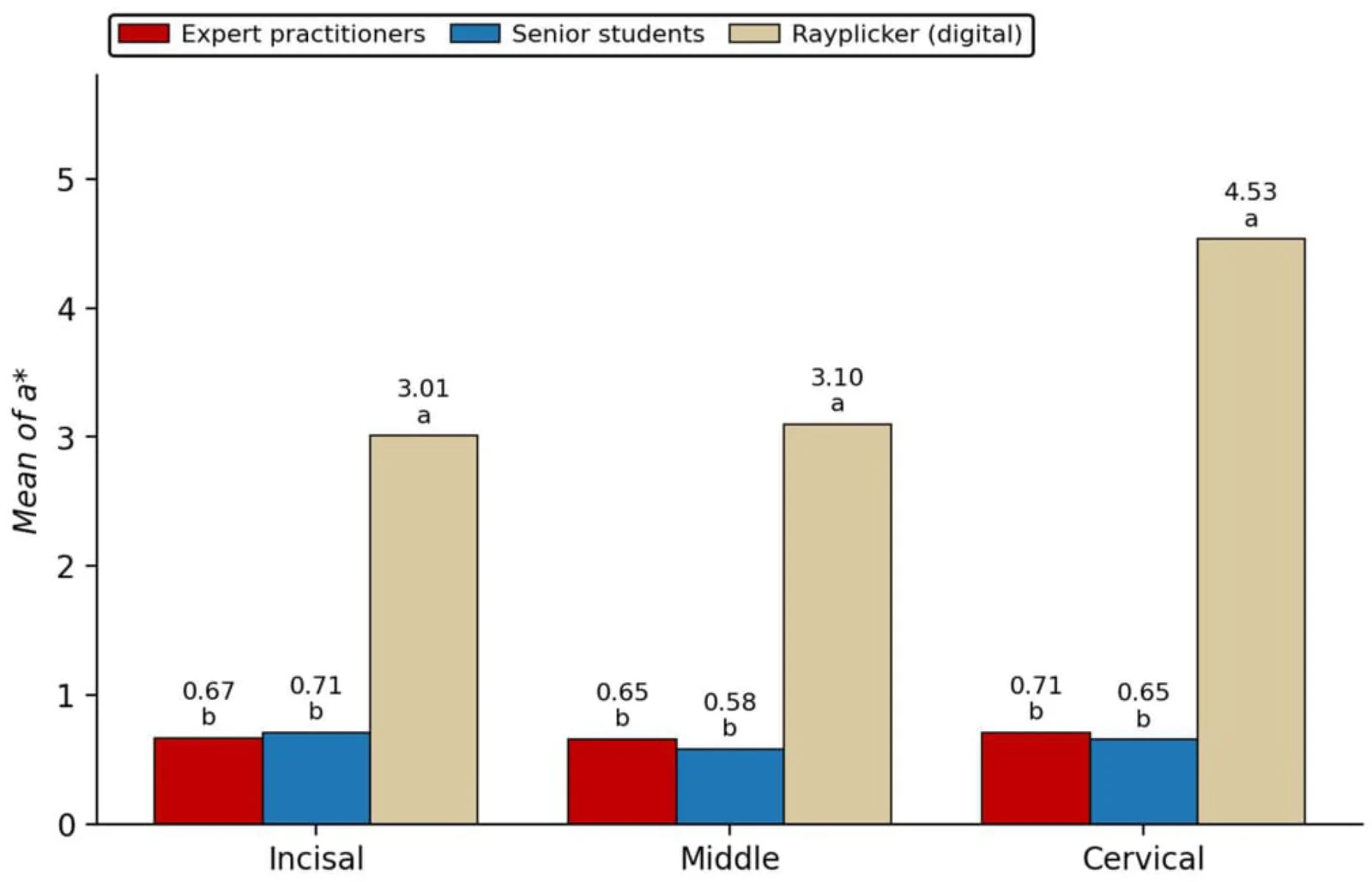
a* Comparison between Expert practitioners, senior students and a* in each location (*n* = 40). Different superscript letters above bars indicate statistically significant differences between groups (Dunn’s post hoc test), *p* ≤ 0.05.

Regarding a* values, representing the red–green axis, the Rayplicker Handy recorded significantly higher a* values (more reddish shades) than both expert practitioners and senior students across all three tooth thirds (*p* < 0.001; Figure 3). No significant differences were found between the two visual examiner groups (*p* > 0.05), demonstrating consistent human perception along this color axis. The Friedman test confirmed significant variation among methods in all regions (*p* < 0.001), and Dunn’s post hoc test verified that a* values obtained with Rayplicker were significantly higher than those from both human groups (*p* < 0.001).

**Figure 4:**
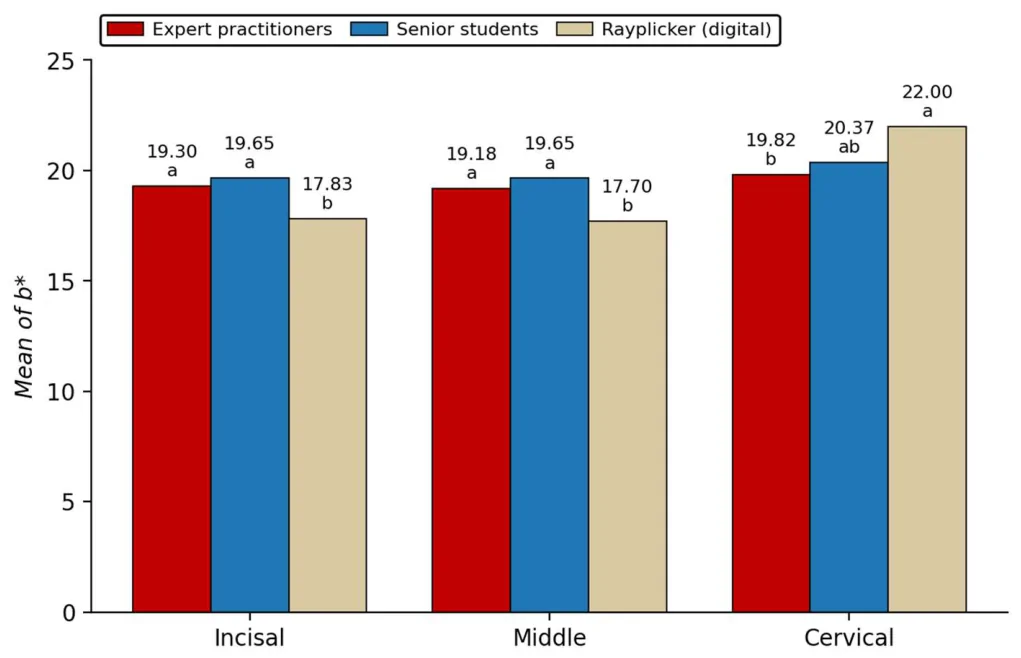
b* Comparison between expert practitioner, senior students and b* in each location (*n* = 40). Different letters above bars indicate statistically significant differences between groups (Dunn’s post hoc test), *p* ≤ 0.05.

For b* values, representing the yellow–blue axis, significant differences among the three methods were observed in all tooth thirds (Friedman test, *p* < 0.001 in the incisal and middle thirds and *p* = 0.005 in the cervical third; Figure 4). In the middle third, the Rayplicker Handy recorded significantly lower b* values, indicating a greater tendency towards blue, compared to both examiner groups (*p* < 0.001). In the incisal third, this difference was also significant for both groups, though less pronounced against expert practitioners (*p* = 0.037) than against students (*p* < 0.001). In the cervical third, Rayplicker instead showed significantly higher b* values (mean = 22.00 ± 5.69), corresponding to a more yellow hue, than experts (*p* = 0.005), while the difference between students and Rayplicker was not significant (*p* = 0.644). No significant difference between expert practitioners and senior students was found in any tooth third for b* (*p* > 0.05), consistent with the L* and a* findings.

**Figure 5:**
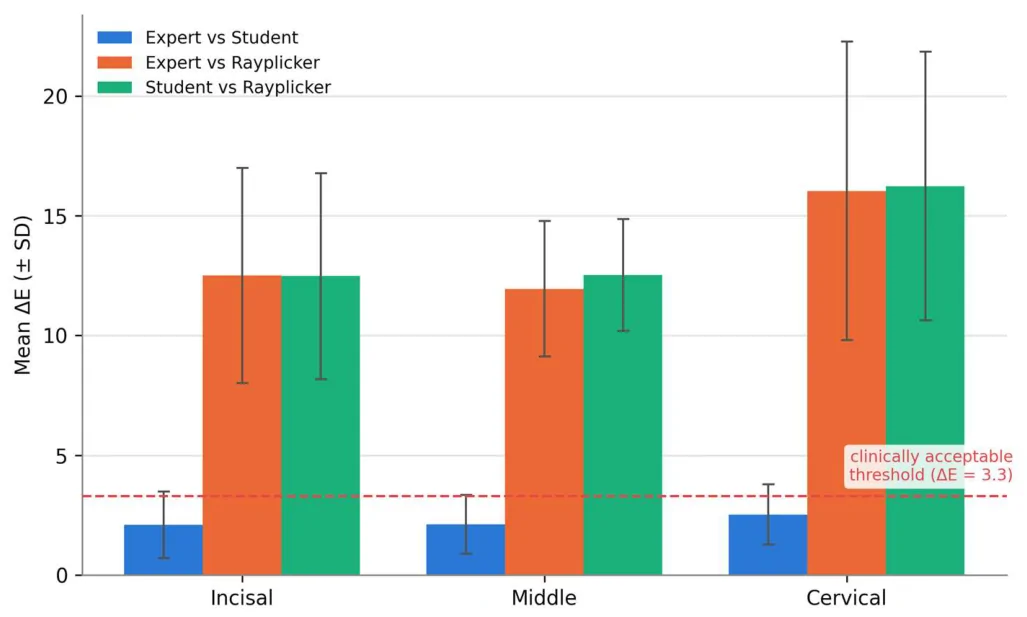
Mean ΔE*ab (±SD) between shade-matching methods, by tooth region (*n* = 40).

To directly quantify agreement between methods, colour differences (ΔE*ab, calculated as the Euclidean distance in CIELAB space, ΔE*ab = √(ΔL*^2^ + Δa*^2^ + Δb*^2^)) were computed between the expert-practitioner average, the senior-student average, and the Rayplicker reading for each participant and tooth region (Table 3, Figure 5). The mean ΔE*ab between expert practitioners and senior students was small in every region (1.9–2.5), above the 50:50% perceptibility threshold (ΔE*ab = 1.2) but within the 50:50% acceptability threshold (ΔE*ab = 2.7), and did not differ significantly from region to region. In contrast, both the expert–Rayplicker and student–Rayplicker ΔE*ab values were far larger (approximately 12–16 across regions), consistently exceeding both thresholds, and were significantly greater than the expert–student ΔE*ab in every region (Friedman test, *p* < 0.001, with Dunn–Nemenyi post hoc comparisons). The expert–Rayplicker and student–Rayplicker ΔE*ab values were not significantly different from one another in any region (*p* > 0.05), indicating that both examiner groups deviated from the digital reference to a similar degree.

**Figure 6:**
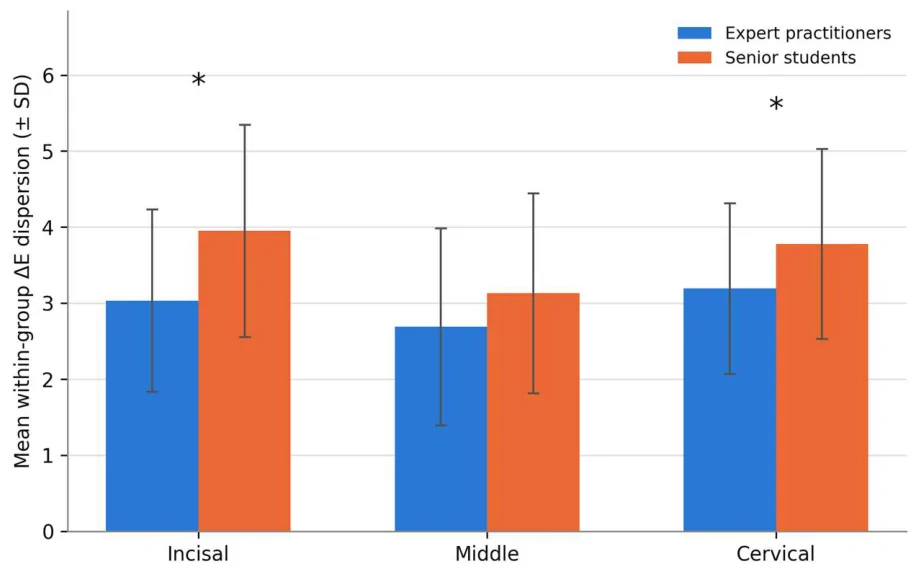
Inter-examiner within-group dispersion (mean ΔE*ab ± SD) by tooth region (*n* = 40). * Statistically significant difference between groups (Wilcoxon signed-rank test), *p* ≤ 0.05.

Because the study objective also called for assessing inter-examiner consistency within each group, the dispersion of the five raters around their group mean was calculated for every participant and region (mean ΔE*ab of each individual examiner to the group centroid; Table 4, Figure 6). Expert practitioners showed lower within-group dispersion than senior students in all three regions, and this difference reached statistical significance in the incisal (Wilcoxon signed-rank test, *p* < 0.001) and cervical thirds (*p* = 0.049), but not in the middle third (*p* = 0.116). This indicates that, although the two groups selected similar shades on average (Table 3), expert practitioners agreed with one another more closely than senior students did, i.e., clinical experience was associated with greater reproducibility among raters rather than with a difference in average agreement with the digital reference. Repeated same-examiner measurements were not collected in this study, so true intra-examiner (test–retest) reliability could not be calculated; this is addressed in the Limitations. After applying a Holm–Bonferroni correction across the three regional comparisons, the incisal result remained statistically significant, whereas the cervical result no longer reached significance.

In this sensitivity analysis, the group effect on inter-examiner dispersion remained statistically significant in all three tooth regions after accounting for participant- and examiner-level clustering (Table 5). This remained significant across all three regions after the same Holm–Bonferroni correction.

As a sensitivity analysis, the primary CIE L*a*b* comparison (Table 2) and the inter-examiner within-group dispersion analysis (Table 4) were repeated using only complete-case observations, excluding all 24 corrected or excluded readings. The significance pattern was unchanged for every comparison (Table 6), confirming that the data-correction procedure did not materially alter the study’s conclusions [34].

As a complementary reliability statistic, intraclass correlation coefficients were calculated for each region and CIE L*a*b* component within each examiner group (Table 7). Absolute-agreement ICC(A,1) values were directionally higher for expert practitioners than for senior students in all nine region-by-component comparisons (range: 0.22–0.35 for experts versus 0.04–0.20 for students), but the 95% confidence intervals overlapped between groups in every comparison, so this analysis corroborates rather than independently establishes the between-group difference. By conventional benchmarks, reliability was poor for most comparisons and did not exceed the moderate range for either group. For senior students specifically, the incisal-region ICC did not differ significantly from zero for any of the three CIE L*a*b* components (ICC(A,1) = 0.04–0.07, *p* = 0.056–0.158), indicating that individual student examiners’ incisal readings could not be distinguished from random variation around the group mean; expert practitioners retained statistically significant, if modest, reliability in the incisal region (ICC(A,1) = 0.24–0.25, *p* < 0.001).

## 4. Discussion

Expert practitioners and senior dental students performed similarly when using the visual method (Vita 3D-Master guide), with no statistically significant difference in the mean ΔE*ab values. This suggests that clinical experience did not significantly change the closeness of shade selection to that of less experienced students when relying on visual assessment alone. The outcome aligns with earlier research indicating that visual shade selection is affected by multiple factors such as ambient light, observer fatigue, and individual color perception [35]. However, when the internal (inter-examiner) within-group dispersion of each group was compared, expert practitioners showed significantly lower dispersion (closer within-group agreement) than senior students in the incisal (*p* < 0.001) and cervical (*p* = 0.049) thirds, with no significant difference in the middle third (*p* = 0.116). The cervical third showed the greatest dispersion for both groups; this region has a higher chroma and lower lightness, which may make visual matching more challenging [36]. This indicates that, while clinical experience did not bring visual shade selections closer to the students’ selections on average, it was associated with more reproducible (though not necessarily more accurate) shade choices among examiners.

Within-group dispersion and the intraclass correlation coefficient capture related but distinct aspects of inter-examiner agreement, and reporting both is consistent with recommended practice in multi-rater clinical measurement research, where reliance on a single statistic can understate or overstate agreement depending on the scale, distribution, and clustering structure of the underlying measurements [33,37]. When examined using the intraclass correlation coefficient, absolute agreement was directionally better for expert practitioners than for senior students in every region-by-component comparison, consistent with the within-group dispersion and mixed-model findings above, although the confidence intervals for the two groups overlapped throughout, so this analysis corroborates rather than independently confirms the between-group difference already established by those analyses. Reliability was poor-to-moderate by conventional benchmarks for both groups across most regions, and, among senior students in the incisal region specifically, the ICC did not differ significantly from zero, meaning individual student examiners’ incisal readings could not be distinguished from random variation around the group mean using this metric.

One plausible explanation, in our view, is that visual shade selection with the Vita 3D-Master guide is constrained to a finite, discretely spaced set of shade tabs, so that absolute-agreement statistics—which are highly sensitive to the scale of measurement—may understate true examiner concordance where the underlying measurement scale itself provides only coarse resolution; the within-group ΔE*ab dispersion measure and the Friedman/Wilcoxon comparisons, which assess the data on a continuous and rank basis rather than through strict variance partitioning, may be less sensitive to this quantization effect.

In contrast, the digital method using the Rayplicker handy spectrophotometers produced detailed, systematically different results relative to both groups. It detected color shifts that were not captured visually in all three regions, especially in the cervical area, where more yellow tones were observed. In relation to the visual methods, the digital devices showed lower L* and b* values and higher a* values, highlighting its sensitivity to color variations. These measurements differed systematically from the visual values; because a single trained operator performed all digital measurements in this study, between-operator stability of the digital method could not be assessed and is not directly comparable to the between-examiner dispersion reported for the visual groups (see Limitations) [35,38].

The present study’s inferential claims regarding clinical experience were based on Friedman and Wilcoxon tests applied to participant-level group means, a design that does not explicitly model examiner-level clustering. To address this, a supplementary linear mixed-effects model was fitted with participant and examiner specified as crossed random effects, directly testing whether the between-group difference in inter-examiner dispersion persisted once this clustering was accounted for. The group effect remained statistically significant in all three tooth regions, indicating that the observed difference in within-group dispersion between expert practitioners and senior dental students is not an artifact of the original analytic structure. This remained true after applying the same Holm–Bonferroni correction for the three regional comparisons (adjusted *p* = 0.042, 0.042, and 0.012 for the incisal, middle, and cervical regions, respectively) [32]. Crossed random-effects models of this kind are increasingly used in multi-rater method-comparison research to separate true group-level effects from rater-specific and subject-specific variability [30,31].

Given the statistically significant differences between methods, Rayplicker measurements diverged consistently from visual shade selection by both expert and student groups, reflecting a systematic difference between approaches rather than demonstrated superiority in accuracy. These findings reinforce that digital and visual shade-matching methods are not interchangeable in this study population.

The limitations of visual assessment relative to instrumental methods are well documented: in one clinical study, three independent observers agreed on the same shade in only 26.6% of cases, compared with 83.3% agreement for spectrophotometric assessment of the same teeth, with visually determined shades showing greater color deviation (ΔE*ab) than instrumental measurements in 93.3% of cases [39]. Lighting conditions further affect this gap: under varying ambient light, the repeatability of visual shade selection has been reported at approximately 75%, compared with around 87% for a digital intraoral scanner [35].

The Rayplicker Handy captures the entire tooth surface in a single acquisition and processes the data through spectral imaging into CIELAB L*a*b* values, enabling objective, quantifiable color assessment of the cervical, middle, and incisal thirds that is difficult to achieve reliably with visual shade selection alone [1,14]. Previous studies report excellent device repeatability, with intraclass correlation coefficients above 0.90 (reaching 0.98–0.99 for L* and C*), and a recent systematic review found spectrophotometric devices such as the Rayplicker superior to visual methods in accuracy and consistency [38].

As demonstrated by Hampé-Kautz et al., visual shade determination, despite its widespread use, remains highly subjective and dependent on variables such as the practitioner’s skill and age, the illuminant, and the background [14]. Their in vivo study compared visual shade matching performed by novice and expert practitioners against instrumental methods and found that although the expert achieved matches closer to the Easyshade V spectrophotometer reference than the novice, visual assessment still differed appreciably from instrumental measurement [14]. This persistent variability underscores the inherent limitations of subjective visual assessment, even among trained clinicians. Such limitations have driven the search for more accurate, consistent, and scientific approaches, culminating in the adoption of objective instrumentation such as spectrophotometers, which demonstrate superior precision and accuracy [17,40,41].

Rayplicker was used in this study as a digital comparative reference for evaluating visual shade selections. Reporting results in terms of agreement, concordance, or measurement difference between visual and digital methods is consistent with recent methodological guidance in prosthodontic research by Yeo [42] and with comparable in vivo work evaluating dental shade guides by Ruiz-López et al. [15].

These findings are broadly consistent with other reports comparing shade-matching performance across groups with differing clinical experience. Udiljak et al. evaluated four dental occupational groups (dental technicians, prosthodontic residents, prosthodontic specialists, and dental students) and found closely comparable mean ΔE*ab values (approximately 2.1–2.5 across groups), with no statistically significant difference between groups (*p* > 0.05), concluding that experience level alone did not substantially predict shade-matching accuracy [21]. This closely parallels our expert–student ΔE*ab values (1.9–2.5 across regions, Table 3) and their lack of statistical significance, supporting the interpretation that visual shade-matching agreement relative to a common reference is not strongly determined by formal clinical experience. Regarding inter-examiner within-group dispersion, Knezović Zlatarić et al. reported that even with a calibrated digital spectrophotometer (VITA Easyshade Advance 4.0), inter-observer ΔE*ab among four trained examiners ranged from 3.0 to 5.2, with no statistically significant inter-observer differences (*p* > 0.05) [28], indicating that some measurement variability between examiners persists even with instrumental methods.

Against this benchmark, the within-group ΔE*ab dispersion we observed among expert practitioners (approximately 2.0–2.5 across regions, Table 4) was numerically lower, while the senior students’ dispersion, particularly in the cervical third, approached this instrumental benchmark; this is consistent with clinical calibration in the expert group rather than an artefact of the visual method. Finally, the substantially larger ΔE*ab observed between the visual methods and the Rayplicker in our sample (12–16 across regions) is consistent with reports that agreement between visual and instrumental shade-matching is, at best, moderate: using weighted Cohen’s kappa, Lee and Kim found agreement coefficients of only 0.47–0.50 between visual assessment and spectrophotometric or intraoral-scanner measurements [41], reinforcing that visual and digital methods, while each individually reproducible, are not interchangeable and appear to measure shade along a systematically offset scale.

Beyond measurement-scale quantization (discussed above), several additional factors plausibly contribute to the within-group dispersion observed in this study. Visual shade guides such as the Vita 3D-Master are known to have substantial, non-uniform gaps between adjacent shade tabs, which can preclude an exact match for a given tooth and may add to measured dispersion regardless of examiner skill [15,43]. Individual-level factors also play a role: color perception is influenced by the examiner’s experience, eye fatigue, and age-related changes in the eye, and a minority of individuals have some degree of color vision deficiency that can directly impair shade discrimination [9,44,45,46]. Because all examiners passed Ishihara screening, congenital color vision deficiency is an unlikely explanation for the differences in within-group dispersion. Ambient lighting conditions during shade-matching, including metameric effects in which colors that match under one light source fail to match under another, and natural age-related changes in tooth color itself, add further variability to the shade-matching task independent of examiner-level factors [47,48]. Because the lighting in this study combined clinic lighting with ambient daylight and was not measured objectively, variation in illumination may have contributed to the dispersion of visual selections. These factors would be expected to affect all examiners to some degree, but may disproportionately affect less experienced examiners, consistent with the greater dispersion observed among senior students in this study.

### 4.1. Limitations

This study has some limitations. Only one tooth (the maxillary right central incisor) was assessed, using a single digital reference device (Rayplicker Handy), so the findings may not generalize to other teeth, to other visual shade guides (e.g., VITA classical or the VITA Linearguide 3D-Master), or to other digital devices such as spot-measurement spectrophotometers. Repeated measurements by the same examiner were not collected, so true intra-examiner (test–retest) reliability could not be assessed. Similarly, repeated Rayplicker readings at the same site were not obtained in this study, so the repeatability of the digital device itself could not be directly assessed; this should be evaluated in future work using repeated measurements, consistent with recommended practice for reproducibility assessment of digital shade-matching devices [49]. During data cleaning, cross-referencing each recorded L*a*b* value against its corresponding VITA 3D-Master shade tab identified a small number of transcription errors (18 of 1200 visual readings, ~1.5%, plus six unrecoverable readings for one shade tab); these were corrected or excluded, and Table 2, Table 3 and Table 4, and Figure 2, Figure 3, Figure 4, Figure 5 and Figure 6 all reflect these corrections. No a priori sample-size calculation was performed, and both participants and examiners were convenience samples. The sample was sufficient to detect the large differences between visual and digital methods, but the comparison between experience groups rested on only five examiners per group, which also precluded subgroup analyses (e.g., by gender or years of experience). Findings on clinical experience should therefore be regarded as exploratory and confirmed with larger, independently sampled examiner panels. The expert and student groups also differed in age and in routine volume of shade selection, which could not be separated from clinical experience; differences between the groups should therefore be read as associated with, not caused by, clinical experience. All examiners were female, and reported gender differences in shade-matching performance [50,51] mean the findings may not apply to male examiners. All participants were female dental students aged 20–25 years with intact, unrestored incisors. This homogeneous sample covers a narrow range of tooth shades and excludes the darker, more chromatic, age-related tooth colors of older and general patient populations, to which the findings may not generalize [52]. Finally, visual shade selection was performed under mixed clinic lighting and ambient daylight, and color temperature, illuminance and color rendering were not measured during the sessions, so variation in illumination between sessions cannot be excluded [53].

In addition, Rayplicker was used in this study as a comparative reference rather than a validated, independently verified criterion standard for true tooth colour; differences between visual and digital measurements should therefore be interpreted as disagreement between methods rather than evidence of differential accuracy. Data review and correction were performed by one of the study’s co-authors prior to statistical analysis, blinded to examiner group and study outcome [54]. Flagged readings were corrected to the reference L*a*b* value consistently associated with the recorded shade-tab designation elsewhere in the dataset; the six unrecoverable readings could not be assigned a value with confidence and were excluded, with the corresponding group mean or centroid for that participant and region computed from the remaining examiners in that group (available-case basis).

### 4.2. Clinical Implications

The within-group comparisons confirmed the inherent subjectivity of visual shade selection in both the expert and student groups, although experts showed significantly less inter-examiner dispersion than students in the incisal region after Holm-Bonferroni correction for the three regional comparisons, suggesting that greater clinical experience may be associated with reduced, but not eliminated, subjectivity [3]. This underlines the potential value of integrating digital tools into routine practice—not as a replacement for clinical judgment, but as a complementary aid that may be associated with more standardized shade communication. Objective, quantifiable color data of the kind produced by devices such as the Rayplicker could plausibly support more standardized communication with the dental laboratory and help reduce susceptibility to operator-related error; however, this study did not measure laboratory communication, remake rates, restoration quality, or patient satisfaction, so these remain potential clinical implications warranting future outcome-based research rather than demonstrated findings of this study, consistent with recommended practice to avoid overstating unmeasured outcomes in reporting [55].

Beyond this general rationale, the present findings provide a concrete, quantitative basis for when adjunctive digital verification is likely to be most clinically valuable. Visual shade selections by both expert practitioners and senior students differed from the Rayplicker digital reference by approximately 12–16 ΔE*ab units across all three tooth regions, several times above the 50:50% acceptability threshold (ΔE*ab = 2.7) generally considered clinically acceptable, indicating that unaided visual shade matching—irrespective of examiner experience—carries a substantial risk of a color mismatch large enough to be clinically relevant. Clinical experience improved inter-examiner agreement in the incisal region yet did not reduce the magnitude of this gap to the digital reference, and senior students’ incisal-region judgements could not be statistically distinguished from chance (Table 7). Taken together, these findings suggest that adjunctive digital shade verification may add the greatest clinical value precisely where visual consistency is weakest—among less experienced examiners and in tooth regions, such as the cervical and incisal thirds, where measured dispersion was highest—offering an evidence-based basis for targeting digital verification and shade-matching training within restorative and prosthodontic workflows.

These findings are consistent with recommendations favoring broader adoption of digital shade-matching technologies in clinical settings [38]. Such technologies could plausibly help reduce operator-related error and support more standardized shade communication [35,38], though this study did not measure restoration quality, remake rates, or the aesthetic quality of finished restorations, so these remain potential clinical implications rather than demonstrated outcomes of this work. Exposing undergraduate training and daily dental workflows to such technologies may nonetheless be a reasonable direction for future educational and clinical practice.

## 5. Conclusions

Visual and digital shade selections differed substantially in this study population, exceeding the 50:50% acceptability threshold. Expert practitioners and senior students selected comparably close shades to one another on average, but expert practitioners showed significantly lower within-group dispersion (closer inter-examiner agreement) than senior students in the incisal region after correction for multiple regional comparisons, a finding corroborated across all three tooth regions by a supplementary mixed-effects analysis accounting for examiner-level clustering and directionally supported by a complementary intraclass correlation analysis, indicating that clinical experience was associated with greater reproducibility of visual shade selection even though it did not bring selections closer to the Rayplicker measurements. This highlights the inherent subjectivity of visual shade matching relative to instrumental measurement. By quantifying where, and by how much, visual shade selection diverges from an objective digital reference, this study offers an evidence-based basis for targeting adjunctive digital verification and shade-matching training toward the examiners and tooth regions that need it most. These findings suggest a potential role for digital shade-matching devices in clinical practice and dental education; whether their use translates into enhanced esthetic outcomes, reduced human error, or improved patient satisfaction in restorative and prosthetic dentistry was not evaluated in this study and warrants future outcome-based research.

## Figures and Tables

**Table 1:** Characteristics of the examiner groups.

Characteristic	Expert Practitioners (*n* = 5)	Senior Dental Students (*n* = 5)
Age, years (range)	38–45	20–22
Sex	5 female	5 female
Professional status	Faculty consultants	Final-year dental students
Specialty	Restorative dentistry (1) Prosthodontics (4)	—
Clinical experience, years	13–20	None (supervised only)
Shade selections per month	≥20	≈4
Prior use of VITA 3D-Master	5/5 (routine clinical use)	5/5 (during clinical training)
Formal shade-matching training	5/5	5/5
Color vision (Ishihara)	Normal (5/5)	Normal (5/5)

**Table 2:** Comparison of CIE Lab values by visual-shade guide among expert practitioners, senior dental students, and digital-shade guide using Rayplicker Handy measurements across tooth regions (*n* = 40).

CIE Lab Values	Tooth Regions	Visual-Shade Guide (Vita 3D Master)		Digital-Shade Guide (Rayplicker)	*p*
		Expert Practitioners	Senior Students		
L*	Incisal				
	Mean ± SD.	79.64 ± 1.97	79.57 ± 2.04	67.83 ± 5.00	<0.001 *
	Median (IQR)	78.78 ^a^ (78.50–80.86)	79.94 ^a^ (77.97–80.95)	68.30 ^b^ (64.40–70.55)	
	Sig. bet.	*p*1 = 0.973, *p*2 = <0.001 *, *p*3 = <0.001 *			
	Middle				
	Mean ± SD.	79.71 ± 2.02	80.15 ± 1.74	68.40 ± 3.43	<0.001 *
	Median (IQR)	78.85 ^a^ (78.66–80.86)	79.77 ^a^ (78.88–81.57)	68.30 ^b^ (68.30–68.30)	
	Sig. bet.	*p*1 = 0.573, *p*2 = <0.001 *, *p*3 = <0.001 *			
	Cervical				
	Mean ± SD.	79.49 ± 2.09	79.84 ± 1.73	65.05 ± 4.95	<0.001 *
	Median (IQR)	78.90 ^a^ (78.34–80.74)	79.92 ^a^ (78.88–81.02)	65.10 ^b^ (64.40–65.90)	
	Sig. bet.	*p*1 = 0.503, *p*2 = <0.001 *, *p*3 = <0.001 *			
a*	Incisal				
	Mean ± SD.	0.67 ± 0.40	0.71 ± 0.48	3.01 ± 1.24	<0.001 *
	Median (IQR)	0.82 ^b^ (0.42–0.91)	0.62 ^b^ (0.40–1.04)	2.90 ^a^ (1.80–4.20)	
	Sig. bet.	*p*1 = 0.896, *p*2 = <0.001 *, *p*3 = <0.001 *			
	Middle				
	Mean ± SD.	0.65 ± 0.41	0.58 ± 0.36	3.10 ± 0.96	<0.001 *
	Median (IQR)	0.82 ^b^ (0.42–0.90)	0.67 ^b^ (0.27–0.84)	2.90 ^a^ (2.90–3.83)	
	Sig. bet.	*p*1 = 0.573, *p*2 = <0.001 *, *p*3 = <0.001 *			
	Cervical				
	Mean ± SD.	0.71 ± 0.44	0.65 ± 0.39	4.53 ± 1.37	<0.001 *
	Median (IQR)	0.80 ^b^ (0.44–0.95)	0.60 ^b^ (0.38–0.87)	4.20 ^a^ (4.20–5.70)	
	Sig. bet.	*p*1 = 0.896, *p*2 = <0.001 *, *p*3 = <0.001 *			
b*	Incisal				
	Mean ± SD.	19.30 ± 2.14	19.65 ± 1.77	17.83 ± 2.18	<0.001 *
	Median (IQR)	18.98 ^a^ (18.20–20.14)	19.59 ^a^ (18.47–20.55)	17.00 ^b^ (16.90–19.40)	
	Sig. bet.	*p*1 = 0.173, *p*2 = 0.037 *, *p*3 = <0.001 *			
	Middle				
	Mean ± SD.	19.18 ± 2.09	19.65 ± 1.82	17.70 ± 2.28	<0.001 *
	Median (IQR)	19.24 ^a^ (18.20–19.90)	19.68 ^a^ (18.30–20.62)	17.00 ^b^ (17.00–19.40)	
	Sig. bet.	*p*1 = 0.435, *p*2 = <0.001 *, *p*3 = <0.001 *			
	Cervical				
	Mean ± SD.	19.82 ± 2.32	20.37 ± 2.24	22.00 ± 5.69	0.005 *
	Median (IQR)	19.26 ^b^ (18.50–21.49)	20.27 ^ab^ (19.20–21.73)	19.40 ^a^ (19.40–26.30)	
	Sig. bet.	*p*1 = 0.065, *p*2 = 0.005 *, *p*3 = 0.644			

*p*: *p* value for Friedman test (comparison among the three methods). *p*1: *p* value for experts vs. students. *p*2: *p* value for experts vs. Rayplicker. *p*3: *p* value for students vs. Rayplicker. IQR: interquartile range; SD: standard deviation. Different superscript letters indicate statistically significant differences in medians (Dunn’s post hoc test), *p* ≤ 0.05. * Statistically significant at *p* ≤ 0.05.

**Table 3:** Mean ΔE*ab (CIELAB) between visual-shade selection (expert practitioners, senior students) and the Rayplicker Handy digital measurement, by tooth region (*n* = 40).

Tooth Region		Expert vs. Student	Expert vs. Rayplicker	Student vs. Rayplicker	*p* (Friedman)
Incisal	Mean ± SD	2.09 ± 1.39 ^a^	12.50 ± 4.49 ^b^	12.47 ± 4.31 ^b^	<0.001 *
	Median (IQR)	1.97 (1.26–2.89)	13.73 (10.68–14.66)	12.97 (9.38–14.14)	
	Sig. bet.	*p*1 = <0.001 *, *p*2 = <0.001 *, *p*3 = 0.940	*p*1 = <0.001 *, *p*2 = <0.001 *, *p*3 = 0.940	*p*1 = <0.001 *, *p*2 = <0.001 *, *p*3 = 0.940	
Middle	Mean ± SD	2.11 ± 1.23 ^a^	11.94 ± 2.83 ^b^	12.52 ± 2.33 ^b^	<0.001 *
	Median (IQR)	1.93 (1.10–2.54)	12.03 (10.88–13.95)	12.64 (11.86–14.13)	
	Sig. bet.	*p*1 = <0.001 *, *p*2 = <0.001 *, *p*3 = 0.314	*p*1 = <0.001 *, *p*2 = <0.001 *, *p*3 = 0.314	*p*1 = <0.001 *, *p*2 = <0.001 *, *p*3 = 0.314	
Cervical	Mean ± SD	2.52 ± 1.25 ^a^	16.03 ± 6.23 ^b^	16.24 ± 5.60 ^b^	<0.001 *
	Median (IQR)	2.57 (1.61–3.51)	15.86 (13.72–17.14)	15.68 (14.01–17.87)	
	Sig. bet.	*p*1 = <0.001 *, *p*2 = <0.001 *, *p*3 = 0.896	*p*1 = <0.001 *, *p*2 = <0.001 *, *p*3 = 0.896	*p*1 = <0.001 *, *p*2 = <0.001 *, *p*3 = 0.896	

ΔE*ab: colour difference (Euclidean CIELAB distance). *p*: *p* value for Friedman test (comparison among the three ΔE*ab series). *p*1: expert-vs.-student ΔE*ab vs. expert-vs-Rayplicker ΔE*ab. *p*2: expert-vs.-student ΔE*ab vs. student-vs.-Rayplicker ΔE*ab. *p*3: expert-vs.-Rayplicker ΔE*ab vs. student-vs.-Rayplicker ΔE*ab (Dunn–Nemenyi post hoc). IQR: interquartile range; SD: standard deviation. Different superscript letters (a, b) indicate statistically significant post hoc differences between columns, *p* ≤ 0.05. * Statistically significant at *p* ≤ 0.05.

**Table 4:** Inter-examiner within-group dispersion (mean ΔE*ab of the five raters around their group mean) for expert practitioners and senior students, by tooth region (*n* = 40).

Tooth Region		Expert Practitioners	Senior Students	*p* (Wilcoxon)
**Incisal**	Mean ± SD	3.03 ± 1.20	3.95 ± 1.40	<0.001 *
	Median	3.04	3.66	
**Middle**	Mean ± SD	2.69 ± 1.30	3.13 ± 1.32	0.116
	Median	2.65	3.39	
**Cervical**	Mean ± SD	3.19 ± 1.12	3.78 ± 1.25	0.049 *
	Median	3.03	3.66	

Values are the mean ΔE*ab of each individual rater to their group’s mean L*a*b* per participant (lower = less dispersion). *p*: Wilcoxon signed-rank test (paired by participant). * Statistically significant at *p* ≤ 0.05.

**Table 5:** Supplementary crossed-effects linear mixed-model analysis of inter-examiner dispersion, confirming the group (expert/student) effect after accounting for participant- and examiner-level clustering, by tooth region (*n* = 40 participants; 10 examiners).

Tooth Region	Coefficient (Student vs. Expert), ΔE*ab	SE	95% CI	*p*
Incisal	0.92	0.41	0.11, 1.72	0.026 *
Middle	0.43	0.19	0.07, 0.80	0.021 *
Cervical	0.61	0.21	0.19, 1.02	0.004 *

Linear mixed-effects model (REML) of each rater’s ΔE*ab dispersion from their own group’s centroid, with participant and examiner specified as crossed random effects and group as a fixed effect (reference: expert practitioners). Positive coefficients indicate greater within-group dispersion among senior dental students relative to expert practitioners. * Statistically significant (*p* < 0.05).

**Table 6:** Sensitivity analysis comparing the main (corrected-data) analysis with a complete-case analysis excluding all corrected or excluded readings.

Analysis	Region	Main Analysis (Corrected Data)	Complete-Case Analysis
CIE L*a*b* values (Friedman test)	Incisal, Middle, Cervical	*p* < 0.05 for L*, a*, and b* in all three regions	*p* < 0.05 for L*, a*, and b* in all three regions
Inter-examiner consistency (Wilcoxon test)	Incisal	*p* < 0.001	*p* < 0.001
	Middle	*p* = 0.116	*p* = 0.118
	Cervical	*p* = 0.049	*p* = 0.049

Complete-case analysis excludes all 24 readings (2.0% of 1200) that required correction or were excluded as unrecoverable (see Limitations); both analyses yield the same pattern of statistical significance.

**Table 7:** Intraclass correlation coefficients (ICC) for inter-examiner reliability by tooth region and CIE L*a*b* component, for expert practitioners and senior students (*n* = 40 participants per group, except where noted).

Group	Region	Component	*n*	ICC(A,1) [95% CI]	ICC(A,k) [95% CI]	*p*
Expert	Incisal	L	40	0.24 [0.11, 0.40]	0.61 [0.38, 0.77]	<0.001
		a	40	0.25 [0.12, 0.42]	0.63 [0.41, 0.78]	<0.001
		b	40	0.25 [0.13, 0.42]	0.63 [0.42, 0.78]	<0.001
	Middle	L	40	0.34 [0.20, 0.51]	0.72 [0.56, 0.84]	<0.001
		a	40	0.35 [0.21, 0.52]	0.73 [0.57, 0.84]	<0.001
		b	40	0.27 [0.14, 0.44]	0.65 [0.45, 0.79]	<0.001
	Cervical	L	40	0.22 [0.10, 0.38]	0.59 [0.34, 0.76]	<0.001
		a	40	0.23 [0.11, 0.40]	0.61 [0.38, 0.77]	<0.001
		b	40	0.32 [0.18, 0.48]	0.70 [0.53, 0.82]	<0.001
Student	Incisal	L	38	0.07 [−0.01, 0.21]	0.29 [−0.08, 0.57]	0.056
		a	38	0.06 [−0.02, 0.20]	0.25 [−0.13, 0.55]	0.088
		b	38	0.04 [−0.03, 0.16]	0.17 [−0.19, 0.48]	0.158
	Middle	L	39	0.20 [0.08, 0.36]	0.56 [0.30, 0.74]	<0.001
		a	39	0.19 [0.07, 0.35]	0.53 [0.27, 0.73]	<0.001
		b	39	0.10 [−0.01, 0.25]	0.35 [−0.03, 0.62]	0.034
	Cervical	L	37	0.13 [0.03, 0.28]	0.44 [0.14, 0.66]	0.002
		a	37	0.09 [0.00, 0.24]	0.34 [0.01, 0.61]	0.022
		b	37	0.13 [0.03, 0.28]	0.43 [0.13, 0.66]	0.003

ICC(A,1): single-measures intraclass correlation coefficient, two-way random-effects model, absolute agreement, computed within each examiner group (5 examiners). ICC(A,k): average-measures form of the same model (k = 5). *n* = number of participants with complete ratings from all five examiners in that group for the indicated region/component; participants with a missing rating in that cell (arising from the excluded unrecoverable readings described in Limitations) were omitted from that specific calculation. Interpretation follows conventional benchmarks for single-measure ICC (<0.50 poor, 0.50–0.75 moderate, 0.75–0.90 good, >0.90 excellent).

## Data Availability

The data presented in this study are available as Supplementary Material accompanying this article.

## Supplementary Materials

The following supporting information can be downloaded at: https://www.mdpi.com/article/10.3390/healthcare14213700/s1.

## References

[1] Rashid F., Farook T.H., Dudley J. (2023). Digital shade matching in dentistry: A systematic review. Dent. J..

[2] Mina R.K.G. (2025). Comparing Traditional and Digital Shade Selection in Dentistry: A Review of Literature. Egypt Dent. J..

[3] Abu-Hussein S., Onbasi Y., Berger L., Troll F., Adler W., Wichmann M., Matta R.E. (2023). Comparison of digital and visual tooth shade selection. Clin. Exp. Dent. Res..

[4] Giachetti L., Sarti C., Cinelli F., Russo D. (2020). Accuracy of digital impressions in fixed prosthodontics: A systematic review of clinical studies. Int. J. Prosthodont..

[5] Angelone F., Ponsiglione A., Ricciardi C., Cesarelli G., Sansone M., Amato F. (2023). Diagnostic Applications of Intraoral Scanners: A Systematic Review. J. Imaging.

[6] Witkowski S., Yajima N.D., Wolkewitz M., Strub J.R. (2012). Reliability of shade selection using an intraoral spectrophotometer. Clin. Oral Investig..

[7] Nakhaei M., Ghanbarzadeh J., Amirinejad S., Alavi S., Rajatihaghi H. (2016). The Influence of Dental Shade Guides and Experience on the Accuracy of Shade Matching. J. Contemp. Dent. Pract..

[8] ten Bosch J.J., Coops J.C. (1995). Tooth color and reflectance as related to light scattering and enamel hardness. J. Dent. Res..

[9] Medeiros J.A., Pacheco O.E., Pérez M.M., Carrillo-Pérez F., Herrera L.J., Della Bona A. (2021). Influence of background color on color perception in dentistry. J. Dent..

[10] Mangano F., Gandolfi A., Luongo G., Logozzo S. (2017). Intraoral scanners in dentistry: A review of the current literature. BMC Oral Health.

[11] Kihara H., Hatakeyama W., Komine F., Takafuji K., Takahashi T., Yokota J., Oriso K., Kondo H. (2020). Accuracy and practicality of intraoral scanner in dentistry: A literature review. J. Prosthodont Res..

[12] Tabatabaian F., Beyabanaki E., Alirezaei P., Epakchi S. (2021). Visual and digital tooth shade selection methods, related effective factors and conditions, and their accuracy and precision: A literature review. J. Esthet. Restor. Dent..

[13] Siqueira R., Galli M., Chen Z., Mendonça G., Meirelles L., Wang H.L., Chan H.L. (2021). Intraoral scanning reduces procedure time and improves patient comfort in fixed prosthodontics and implant dentistry: A systematic review. Clin. Oral Investig..

[14] Hampé-Kautz V., Roman T., Schwob T., Cornuault B., Etienne O. (2024). In-vivo repeatability of three intra-oral spectrophotometers. J. Esthet. Restor. Dent..

[15] Ruiz-López J., Pérez M.M., Lucena C., Pulgar R., López-Torruño G., Tejada-Casado M., Ghinea R. (2022). Visual and instrumental covariance error of two dental shade guides: An in vivo study. Clin. Oral Investig..

[16] Bayindir F., Kuo S., Johnston W.M., Wee A.G. (2007). Coverage error of three conceptually different shade guide systems to vital unrestored dentition. J. Prosthet. Dent..

[17] Paravina R.D., Ghinea R., Herrera L.J., Bona A.A., Igiel C., Linninger M., Sakai M., Takahashi H., Tashkandi E., Mar Perez M.D. (2015). Color difference thresholds in dentistry. J. Esthet. Restor. Dent..

[18] Gómez-Polo C., Gómez-Polo M., Quispe López N., Portillo Muñoz M., Montero J. (2022). 3D Master Toothguide is adequate to subjective shade selection?. Medicina.

[19] Kottner J., Audigé L., Brorson S., Donner A., Gajewski B.J., Hróbjartsson A., Roberts C., Shoukri M., Streiner D.L. (2011). Guidelines for Reporting Reliability and Agreement Studies (GRRAS) were proposed. J. Clin. Epidemiol..

[20] Hoenig J.M., Heisey D.M. (2001). The abuse of power: The pervasive fallacy of power calculations for data analysis. Am. Stat..

[21] Udiljak Ž., Illeš D., Knezović Zlatarić D., Ćelić R. (2018). Effect of clinical experience on the shade matching accuracy in different dental occupational groups. Acta Stomatol. Croat..

[22] Milardović Ortolan S., Peršić S., Čelebić A., Mehulić K. (2013). Comparison of time consumption and color matching results of different dental occupational groups. Int. J. Prosthodont..

[23] Ericsson K.A., Krampe R.T., Tesch-Römer C. (1993). The role of deliberate practice in the acquisition of expert performance. Psychol. Rev..

[24] Çapa N., Malkondu Ö., Kazazoğlu E., Çalıkkocaoğlu S. (2010). Evaluating factors that affect the shade-matching ability of dentists, dental staff members and laypeople. J. Am. Dent. Assoc..

[25] Kalyani P., Subiksha K., Jena A., Shashirekha G., Mohanty S., Sharma G. (2023). Effects of eye dominance on shade matching and color perception among the dentist population. Restor. Dent. Endod..

[26] Borea (2019). Rayplicker™ User Manual.

[27] Hina M., Ali M.S., Pande D., Pandey K.K., Kandwal N., Tasar D., Jadhav M.S., Xavier S. (2024). A Comparative Study to Check the Accuracy of Tooth Shade Selection With Standardized Digital Photographs and a Spectrophotometer. Cureus.

[28] Knezović Zlatarić D., Illeš D., Alajbeg I.Ž., Žagar M. (2016). In vivo evaluations of inter-observer reliability using VITA Easyshade Advance 4.0 dental shade-matching device. Acta Stomatol. Croat..

[29] (2016). Dentistry—Guidance on Colour Measurement.

[30] Li M., Kong X. (2023). Comparing generalized estimating equation and linear mixed effects model for estimating marginal association with bivariate continuous outcomes. Ophthalmic Epidemiol..

[31] Scandola M., Tidoni E. (2024). Reliability and feasibility of linear mixed models in fully crossed experimental designs. Adv. Methods Pract. Psychol. Sci..

[32] Menyhárt O., Győrffy B. (2025). Multiplicity corrections in life sciences: Challenges and consequences. Int. J. Epidemiol..

[33] Mondal D., Vanbelle S., Cassese A., Candel M.J.J.M. (2024). Review of sample size determination methods for the intraclass correlation coefficient in the one-way analysis of variance model. Stat. Methods Med. Res..

[34] Kunselman A.R. (2023). A brief overview of sensitivity analyses. Fertil. Steril..

[35] Jainer A. (2004). Tooth colour: A review of the literature. J. Dent..

[36] Jainer A., Luo W. (2017). Tooth colour and whiteness: A review. J. Dent..

[37] Ahmad M.Z. (2026). Evaluation of interexaminer reliability in visual shade matching: A comparative analysis of Optilume Trueshade and conventional lighting for permanent anterior teeth. Cureus.

[38] Hardan L., Bourgi R., Cuevas-Suárez C.E., Lukomska-Szymanska M., Monjarás-Ávila A.J., Zarow M., Jakubowicz N., Jorquera G., Ashi T., Mancino D. (2022). Novel trends in dental color match using different shade selection methods: A systematic review and meta-analysis. Materials.

[39] Reyes J., Acosta P., Ventura D. (2019). Repeatability of the human eye compared to an intraoral scanner in dental shade matching. Heliyon.

[40] Floriani F., Jurado C.A., Abuhammoud S., Vargas M., Fischer N.G., Rojas-Rueda S., Lopes G.C. (2024). A comparative study of shade-matching reproducibility using an intraoral scanner and a spectrophotometer. Dent. J..

[41] Lee J.H., Kim H.K. (2024). A comparative study of shade-matching performance using intraoral scanner, spectrophotometer, and visual assessment. Sci. Rep..

[42] Yeo I.L. (2026). From accuracy to clinical meaning in prosthodontic research. J. Adv. Prosthodont..

[43] Ruyter I.E., Nilner K., Möller B. (1987). Color stability of dental composite resin materials for crown and bridge veneers. Dent. Mater..

[44] Pecho O.E., Pérez M.M., Ghinea R., Della Bona A. (2017). Influence of gender on visual shade matching in dentistry. J. Esthet. Restor. Dent..

[45] Wee A.G., Meyer A., Wu W., Wichmann C.S. (2016). Lighting conditions used during visual shade matching in private dental offices. J. Prosthet. Dent..

[46] Redwan H.S., Alsaggaf A.U., Hawsawi R.A., Sembawa S.N., Marghalani A. (2025). Color vision deficiency among dental practitioners and its effect on shade matching ability. Rom. J. Oral Rehabil..

[47] Hasegawa A., Ikeda I., Kawaguchi S. (2000). Color and translucency of five natural central incisors. J. Prosthet. Dent..

[48] Watts A., Addy M. (2001). Tooth discolouration and staining: A review of the literature. Br. Dent. J..

[49] Kupke L., Schwahn C., Krey K.F., Ratzmann A. (2025). In vivo reproducibility of tooth shade determination on intraoral scans compared to the dental spectrophotometric method. Clin. Oral Investig..

[50] Haddad H.J., Jakstat H.A., Arnetzl G., Borbely J., Vichi A., Dumfahrt H., Renault P., Corcodel N., Pohlen B., Marada G. (2009). Does gender and experience influence shade matching quality?. J. Dent..

[51] Udiljak Ž., Pezo H., Ćelić R. (2020). Gender-dependent quality of shade matching of dental professionals and students. Acta Stomatol. Croat..

[52] Torné-Durán S. (2026). Colour perception screening and shade-matching performance among undergraduate dental students: An exploratory observational study. BDJ Open.

[53] Jouhar R., Ahmed M.A., Heboyan A., Faheemuddin M., Mosaddad S.A., Ahmed N. (2024). Analysis of shade-matching ability in dental students: A comparative study under clinical and correcting light conditions. BMC Med. Educ..

[54] Pilowsky J.K., Elliott R., Roche M.A. (2024). Data cleaning for clinician researchers: Application and explanation of a data-quality framework. Aust. Crit. Care.

[55] Su N., van der Linden M.W., Faggion C.M., van der Heijden G.J.M.G. (2023). Assessment of spin in the abstracts of randomized controlled trials in dental caries with statistically nonsignificant results for primary outcomes: A methodological study. Caries Res..

